# Nursing management of treatment-related venous thromboembolism in patients with multiple myeloma

**DOI:** 10.3389/fmed.2023.1153694

**Published:** 2023-04-18

**Authors:** Bianhong Yang, Chao Liu, Zeyu Lin, Chuanying Geng, Zhiyao Zhang

**Affiliations:** Department of Hematology, Beijing Chaoyang Hospital, Capital Medical University, Beijing, China

**Keywords:** multiple myeloma, nursing, venous thromboembolism, management, treatment

## Abstract

**Objectives:**

Venous thromboembolism (VTE) is a common complication among patients with newly diagnosed multiple myeloma (NDMM). Therefore, this study aimed to analyze the incidence and risk factors associated with VTE in the current era of thromboprophylaxis and to propose appropriate nursing measures.

**Methods:**

A total of 1,539 NDMM patients were retrospectively analyzed. All patients underwent VTE risk assessment and received aspirin or low molecular weight heparin (LMWH) to prevent thrombosis, followed by appropriate care based on their individual thrombosis risk. The incidence of VTE and its related risk factors were then analyzed.

**Results:**

All patients received at least four cycles of therapy containing immunomodulators (IMiDs) and/or proteasome inhibitors (PIs). We assigned 371 patients (24.1%) to the moderate-risk thrombosis group, who received daily aspirin (75 mg) for thrombosis prevention and 1,168 patients (75.9%) to the high-risk group, who received daily low molecular weight heparin (3,000 IU) for thrombosis prevention two times a day. Among all the patients, 53 (3.4%) experienced lower extremity venous thromboembolism events, with three of those patients experiencing a concurrent pulmonary embolism. A multivariate analysis indicated that bed rest lasting more than 2 months and plasma cells of ≥60% were independent factors associated with thrombosis.

**Conclusion:**

More effective risk assessment models are needed to predict thrombosis accurately. In addition, nurses involved in the treatment and management of thrombosis should continually engage in professional development to enhance their knowledge and skills.

## 1. Introduction

Venous thromboembolism (VTE) is a common complication among patients with multiple myeloma (MM). The cause is associated with increased levels of coagulation-stimulating factors and monoclonal gamma globulin in MM patients, as well as the increased use of immunomodulators (IMiDs) such as thalidomide, lenalidomide, and pomalidomide, and proteasome inhibitors (PI) such as carfilzomib and dexamethasone (1–4).

The prevention of VTE is of utmost importance, and nursing plays an important role in its prevention, even more so than treatment. Clinical research has placed great importance on the prevention of VTE (5). However, nurses' understanding of VTE remains suboptimal, especially in relation to disease-specific and drug-related prevention measures.

Therefore, continuing education for nurses must include comprehensive project studies and practices to strengthen their understanding of deep vein thrombosis, with a specific emphasis on VTE prevention (6, 7). Accurate VTE risk assessment is critical to the development of appropriate preventive measures for MM patients. Based on nursing work for myeloma, we analyzed risk factors associated with VTE during the course of treatment, providing nurses with a foundation for identifying patients at risk for VTE in clinical practice.

Risk assessment models (RAMs) for thrombosis in MM patients typically stratify risk based on algorithms established by organizations such as the International Myeloma Working Group (IMWG) (8), the European Myeloma Network (EMN) (9), and the National Comprehensive Cancer Network (NCCN) (10) risk stratification algorithms and on the selection of thromboprophylaxis in MM patients (Appendix 1) (11). This study retrospectively analyzed 1,539 patients with newly diagnosed MM (NDMM), admitted to Beijing Chaoyang Hospital from 2011 to 2020, who received thromboprophylaxis in accordance with the ethical guidelines established by organizations mentioned above. Among these patients, 53 patients (3.4%) developed VTE during the first four treatment cycles of induction. Based on clinical nursing work, this study aimed to analyze high-risk factors associated with VTE and propose the corresponding nursing measures.

## 2. Methods

### 2.1. Patients

This was a retrospective study. We analyzed the incidence of VTE in all NDMM patients. VTE is diagnosed based on the patient's clinical symptoms, a vascular ultrasound or pulmonary perfusion CT scan, and a D-dimer. The inclusion criteria were as follows: (1) patients with a diagnosis of active MM according to IMWG criteria; (2) patients who received at least four cycles of therapy containing PI (bortezomib, BORT) and/or ImiDs (thalidomide, THAL or lenalidomide, LEN) and dexamethasone (DEX); and (3) patients who agreed to thromboprophylaxis according to the IMWG, EMN, and NCCN risk stratification algorithms (Appendix 1). The exclusion criteria were as follows: (1) patients who had received chemotherapy; (2) patients with relapsed or refractory multiple myeloma (RRMM); (3) patients who had been diagnosed with other active malignant tumors; and (4) patients with a disorder of consciousness or expression. A total of 1,539 NDMM patients who met the inclusion and exclusion criteria and who were treated at Beijing Chaoyang Hospital, Capital Medical University, Beijing, China from 1 January 2011, to 31 December 2020 were enrolled. All patients were followed up for four cycles. This study was approved by the Medical Ethics Committee of Beijing Chaoyang Hospital.

### 2.2. Thrombosis prevention and nursing measures

All patients received thrombosis prevention measures and follow-up in accordance with the guidelines established by organizations such as the International Myeloma Working Group (IMWG), the European Myeloma Network (EMN), and the National Comprehensive Cancer Network (NCCN) (8, 9). These measures included the administration of either aspirin or low molecular weight heparin (LMWH) for thrombosis prevention, as well as related nursing measures. Nursing measures were taken according to the consensus of the Oncology Nursing Society (12). If a patient showed signs or symptoms of VTE during induction therapy, a vascular ultrasound or CT scan was performed, and the patient was re-evaluated for VTE.

### 2.3. Statistical analysis

All data were expressed as median ± standard deviation (SD). A multivariate Cox proportional hazard regression analysis was performed for VTE-related factors, and the results were reported as hazard ratios (HRs) with a 95% confidence interval (95%). SPSS 23.0 software (SPSS Institute) was used for statistical analysis. A *p*-value of < 0.05 was statistically significant, and all tests were bilateral.

## 3. Results

### 3.1. Patients

A total of 1,539 NDMM were retrospectively analyzed. The baseline patient characteristics were as follows: age range of 28–84 years, with a mean age of 59.8 years, male gender was predominant with 897 cases (58.3%) than female gender with 642 cases (41.7%), and the MM subtypes of IgG being 42.9%, IgA being 26.7%, IgD being 8.2%, light chain being 20.4%, and non-secretory being 1.8% of cases. The International Staging System (ISS) was classified as stage I at 15.4%, stage II at 36.2%, and stage III at 48.4%. The Revised International Staging System (R-ISS) stage was stage I in 16.9%, stage II in 56.7%, and stage III in 26.4%. Serum albumin levels were ≥35 g/L in 60.5% of patients and <35 g/L in 39.5%. Serum β2-microglobulin levels were <3.5 mg/L in 25.2% of patients, ≥3.5 mg/L and <5.5 mg/L in 26.4%, and ≥5.5 mg/L in 48.4%. Hemoglobin levels were <100 g/L in 56.5% of patients, serum creatinine levels were ≥177 umol/L in 32.3%, corrected serum calcium levels were ≥2.75 mmol/L in 15.4%, and lactate dehydrogenase (LDH) levels were above the upper limits of normal in 18.4% of patients. Cytogenetic abnormalities by FISH included *t*_(4;14)_ in 18.2% of patients, *t*_(11;14)_ in 16.7%, *t*_(14;16)_ in 2.8%, Del (17p) in 8.5%, and 1q21 gain or 1q21 amplification in 48.8%.

All of the patients received at least four cycles of treatment as part of one of the following regimens: BORT-LEN-DEX (BORT 1.3 mg/m^2^, d1, 4, 8,11; LEN 25 mg, d1–21; DEX 20 mg, d1, 2, 4, 5, 8, 9, 11, 12; 21 d/cycle); BORT-THAL-DEX (BORT 1.3 mg/m^2^, d1, 4, 8,11; THAL 100 mg, d1–21; DEX 20 mg, d1, 2, 4, 5, 8, 9, 11, 12; 21 d/cycle); BORT-Cyclophosphamide (CTX)-DEX (BORT 1.3 mg/m^2^, d1, 4, 8, 11; CTX 300 mg/m^2^, d1–4; DEX 20 mg, d1, 2, 4, 5, 8, 9, 11, 12; 21 d/cycle); BORT-DEX (BORT 1.3 mg/m^2^, d1, 4, 8, 11; DEX 20 mg, d1, 2, 4, 5, 8, 9, 11, 12; 21 d/cycle); and LEN-DEX (LEN 25 mg, d1–21; DEX 20 mg, d1, 2, 8, 9, 15, 16, 22, 23; 28 d/cycle). BORT-LEN-DEX, BORT-THAL-DEX, or BORT-CTX-DEX was used for the treatment of autologous transplant-eligible patients, and BORT-DEX or LEN-DEX was used for the treatment of transplant-non-eligible patients. A total of 826 patients (53.7%) received BORT-LEN-DEX treatment, 161 patients (10.5%) received BORT-THAL-DEX, 257 patients (16.7%) received BORT-CTX-DEX, 187 patients (12.1%) received BORT-DEX, and 108 patients (7%) received LEN-DEX. The selection of the regimens was based on age, organ function, and geriatric assessment (GA) score.

### 3.2. Venous thromboembolism prophylaxis and events

Before the induction treatment, all of the patients underwent the thrombus risk assessment according to the RAM (Appendix 1). The VTE prophylaxis adapted the IMWG, EMN, and NCCN risk stratification algorithms (8–10). In total, 371 patients (24.1%) were assessed as having intermediate risk and received thromboprophylaxis with aspirin at a dose of 75 mg per day, 1,168 patients (75.9%) were assessed as having high risk and received thromboprophylaxis with LMWH at a dose of 3,000 IU, administered every 12 h. Even though 53 VTE events (3.4%) occurred, all of the venous thrombosis occurred in the lower limbs. Among them, three patients issued pulmonary embolisms at the same time. All of the VTE events occurred during the first two cycles of induction treatment. The 53 patients were at a high risk of RAM and were treated with low-dose LMWH (3,000 IU, q12h) to prevent thrombosis. When thrombosis occurred, the dose of LMWH was increased to 6,000 IU, q12h. After 2 weeks of LMWH treatment, the thrombus disappeared, including the pulmonary embolism. No patient died due to the thrombus.

### 3.3. The role of nurses

The patient's performance status was one factor in thromboembolism risk. In the prevention of thromboembolism, the nurse's role was to educate patients and their caregivers on the patient's risk factors as well as signs and symptoms, including pain, swelling, erythema, and/or warmth of the affected extremity (12–14). Venous lower limb thrombosis may be traveling from an extremity into the lung, resulting in a pulmonary embolism. Signs and symptoms of pulmonary embolism include tachypnea, difficulty breathing, cough, hemoptysis, pleuritic pain, and cyanosis. The nurse must routinely monitor for specific signs and symptoms and seek immediate medical care if any of those signs or symptoms appears.

For MM patients experiencing bone involvement or compression fractures, bone pain is a major complaint. Nurses should encourage patients to roll over every 2 h to avoid pressure sores and lower limb thrombosis. If the patient could not roll over due to the pain, the patient was asked to lie on an air cushion and use an antithrombotic pressure pump in the lower limbs.

### 3.4. High-risk factors for thrombosis

Despite the aforementioned measures, 53 patients (3.4%) developed venous thrombosis, and three of the patients experienced pulmonary embolisms. All 53 venous thrombosis events occurred during the first two cycles of treatment. The univariate analysis revealed that staying in bed for more than 2 months, a pelvis fracture, diabetes mellitus, obesity (Body mass index >25), plasma cells ≥60%, and accepting IMiDs in combined chemotherapy were the high-risk factors for thrombosis. Multivariate analysis suggests that, by staying in bed for more than 2 months, having plasma cells of ≥60% was the independent related factor of thrombosis (Table 1). Nurses should pay more attention to this kind of patient.

**Table 1:** Risk factors of thrombosis of newly diagnosed multiple myeloma under the thromboprophylaxis according to the risk assessment model (RAM).

**Clinical parameters**	**Univariate analysis**		**Multivariate analysis**	
	**OR (95% CI)**	***P***	**OR (95% CI)**	***P***
Gender: Male vs. female	1:0.42 (0.12–1.21)	0.097	1:0.52 (0.22–1.34)	0.121
Age: ≤ 60 vs. >60 years	1:1.21 (0.78–4.23)	0.833	1:1.08 (0.66–3.47)	0.845
Subtype: IgG vs. IgA vs. IgD vs. light chain vs. non-secretory	1:1.12:1.24:1.01:0.85 (0.88–1.43)	0.927	1:1.03:1.28:0.98:0.79 (0.91–2.08)	0.876
ISS stage: I vs. II vs. III	1:1.12:2.03 (0.56–2.51)	0.241	1:1.99:2.41 (0.49–2.23)	0.351
Hemoglobin: ≥100 vs. < 100 g/L	1:0.92 (0.45–3.73)	0.879	1:1.12 (0.44–3.71)	0.876
Serum albumin: ≥35 vs. < 35 g/L	1:1.83 (0.38–5.73)	0.078	1:2.15 (0.32–4.29)	0.054
Serum β2-microglobulin: < 35 g/L vs. ≥3.5 mg/L and < 5.5 vs. ≥5.5 mg/L	1:1.21:1.53 (0.99–1.87)	0.878	1:1.34:2.01 (0.78–2.56)	0.128
Serum creatinine: < 177 vs. ≥177 umol/L	1:2.31 (0.32–3.12)	0.211	1:2.13 (0.44–3.01)	0.062
Cytogenetic abnormalities by FISH: Standard risk vs. high risk	1:1.17 (0.17–1.43)	0.177	1:1.29 (0.21–1.44)	0.157
Serum calcium: < 2.75 vs. ≥2.75 mmol/L	1:2.03 (0.86–5.46)	0.075	1:1.98 (0.81–3.20)	0.129
Lactate dehydrogenase (LDH): ≤ ULN vs. >ULN	1:2.58 (0.22–6.51)	0.062	1:2.34 (0.32–5.42)	0.075
Stay in bed: ≤ 2 vs. >2 months	1:4.27 (1.21–7.82)	0.011	1:4.67 (1.44–7.54)	0.002
Pelvis fracture: No vs. yes	1:2.69 (2.11–6.10)	0.047	1:2.54 (2.00–5.43)	0.098
Diabetes mellitus: No vs. yes	1:3.58 (0.89–6.20)	0.039	1:2.38 (0.81–4.22)	0.075
Obesity: BMI ≤ 25 vs. >25	1:2.59 (0.15–1.21)	0.042	1:1.72 (0.24–1.34)	0.219
Plasma cells in bone marrow: < 60% vs. ≥ 60%	1:3.62 (1.23–7.01)	0.020	1:4.02 (1.11–7.21)	0.039
MiDs in combined chemotherapy: No vs. yes	1:4.58 (1.51–4.32)	0.041	1:2.45 (1.31–4.98)	0.066

Cytogenetic abnormalities by FISH: risk stratification according to mSMART 3.0.

ULN, Upper limit of normal; BMI, Body mass index.

## 4. Discussion

Multiple myeloma is one of the most common hematologic malignancies in China. Hypercoagulation in MM patients due to hyperimmunoglobulinemia, combined with the use of conventional therapeutic drugs such as immunomodulators and high-dose dexamethasone and immobilization due to surgery or pain, increases the possibility of venous thromboembolism occurring more in MM patients than in other tumor patients. The incidence range of VTE in MM patients treated with IMID-based combination chemotherapy without preventive intervention was as high as 10–34%. The occurrence of VTE not only restricts the choice of drugs but also seriously affects patients' quality of life. In severe cases, it may cause disability or even threaten the lives of patients.

Of all the 1,539 patients, 53 (3.4%) had a VTE event. All 53 patients received low molecular weight heparin (3,000 IU, q12h) for VTE prevention and routine nursing. Most patients developed pelvic fractures and bone pain, and some patients developed tumor lysis syndrome due to a high tumor burden. The multivariate analysis showed that bed rest for more than 2 months and plasma cells of ≥60% were independent factors associated with fracture and hemolysis-associated thrombosis.

For the 1,539 NDMM patients in our study, we assessed the risk of thrombosis and provided corresponding treatment according to the thrombosis risk stratification. Every patient accepted baseline risk stratification and the use of aspirin for low-risk patients and LMWH as a prophylactic dose for higher-risk patients. Even though 53 (3.4%) VTE events occurred, all 53 patients received low molecular weight heparin (3,000 IU, q12h) for VTE prevention and routine nursing. Most patients developed pelvic fractures and bone pain, and some patients developed tumor lysis syndrome due to a high tumor burden. A multivariate analysis showed that bed rest for more than 2 months and plasma cells of ≥60% were independent factors associated with fracture and hemolysis-associated thrombosis. The results might indicate that the guidelines still have limited power for VTE risk stratification and that more accurate RAM is needed (15).

In recent years, there have been several clinical scores for thrombosis risk stratification in MM patients. The IMPEDE VTE score (Appendix 2) was a widely applied risk prediction tool for VTE in MM (16), which was developed by Fotiou et al. (11) and validated by a series of clinical studies in MM patients (17). We used the IMPEDE VTE score to retrospectively evaluate the risk of thrombosis in these 53 patients. All of these patients were at high risk (>8 scores), even with prophylactic LMWH (-3 score), but if these patients received the therapeutic dose of LMWH, their risk of thrombosis would be reduced to intermediate-risk (score of 4–7). It has been shown that the IMPEDE VTE score may be better than the others for predicting thrombus risk.

In addition, three of the 53 patients suffered a pulmonary embolism and venous thromboembolism of the lower limb. The three patients were men and were accompanied by diabetes, coronary heart disease, and a pelvis fracture; they should be evaluated as high-risk (12 scores) for VTE according to the IMPEDE VTE score. At the very early stage of the newly acquired VTE, thrombosis is often unstable and easy to fall off and enter the pulmonary artery, leading to a pulmonary embolism. Doctors generally might adjust the preventive dose of LMWH to the therapeutic dose of LMWH when the VTE occurs in the lower limb. Nurses should take an active role by educating the patient to reduce lower limb movement to avoid thrombus shedding and closely monitoring the patient's breathing. Nurses should always be concerned about preventing the patient from getting out of bed to prevent compression of blood vessels, properly elevating the limbs to promote reflex, using pressure circulation to drive the pump, or asking the patient to wear lower limb socks to improve edema symptoms to avoid pulmonary embolism, which can be accompanied by chest tightness, shortness of breath, difficulty breathing, and even respiratory arrest leading to death. Once the pulmonary embolism symptoms occur, it is important to notify the doctor immediately and provide appropriate treatment.

In patients with IMiDs, glucocorticoid-based therapy is recommended for the concurrent prevention of VTE. The risk of VTE is highest during the first six cycles of induction therapy due to the greater tumor burden and the release of procoagulant factors by tumor cell apoptosis. At this time, nurses must be aware of potential VTE complications, including pulmonary embolism, evaluate patients according to RAM, and prevent thrombosis. After 6 months, the risk of VTE is relatively low, and prophylactic regimens can be adjusted according to the treatment response of MM patients. It is important to note that, although the incidence of VTE in China is relatively lower than in the United States and Europe, each NDMM should receive RAM scoring to prevent the risk of thrombosis. In summary, as the main caregivers of hospitalized patients, nurses' knowledge of VTE is the key to preventing VTE. Nurses' long-term and repeated use of risk assessment forms can enhance their mastery of VTE-related knowledge. In-depth and standardized VTE training should be carried out according to the characteristics of clinical nurses and school students and the weak areas of VTE knowledge so as to improve the VTE prevention ability of nurses (18–20).

## 5. Conclusion

Nursing is important in preventing VTE in patients with NDMM, the risk of thrombosis should be assessed for each patient, and appropriate measures must be implemented. Although a small number of patients still develop VTE, especially pulmonary embolism, this suggests that existing risk stratification algorithms are limited in their ability to stratify the risk of VTE and that more effective risk assessment models are needed. In addition, the IMPEDE VTE score is a VTE scoring method developed in recent years that may have better results for predicting the risk of thrombosis.

## Data availability statement

The original contributions presented in the study are included in the article/Supplementary material, further inquiries can be directed to the corresponding author.

## Author contributions

BY and CL collected data and completed data analysis. CG and ZL completed the patient condition analysis. ZZ wrote the article. All authors have reviewed the article. All authors contributed to the article and approved the submitted version.
