# Supplementary material for: Nursing management of treatment-related venous thromboembolism in patients with multiple myeloma
Source: Front Med (Lausanne). 2023 Apr 18;10:1153694. doi: 10.3389/fmed.2023.1153694 (PMC10151651; doi:10.3389/fmed.2023.1153694)
Supplement: Supplementary file 1 [file Table_1.pdf]

Appendix 1 International Myeloma Working Group, European Myeloma Network, and National Comprehensive Cancer Network risk stratification algorithm and choice of thromboprophylaxis in patients with multiple myeloma.

Algorithm for MM Patient Risk Stratification		
Patient-related risk factors assign 1 point for each of the below	Disease-related risk factors: assign 1 point for each of the below	Treatment-related risk factors: assign points as seen below
Body mass index >25	Diagnosis of multiple myeloma	IMiD in combination with low-dose dexamethasone (<480 mg/month) *(1 point)*
Age >75	Evidence of hyperviscosity	IMiD plus high-dose dexamethasone (>480 mg/month) or doxorubicin or multiagent chemotherapy (*2 points)*
Personal or family history of VTE		IMiD alone (*1 point)*
Central venous catheter		Erythropoietin use (*1 point)*
Acute infection or Hospitalization		
Blood clotting disorders or Thrombophilia		
Immobility with a performance status of >1		
Comorbidities (liver, renal impairment, chronic obstructive pulmonary disorder, diabetes mellitus, chronic inflammatory bowel disease)		
Race (Caucasian is a risk factor)		
Risk stratification and recommended thromboprophylaxis:<br>0 points: Low risk<br>None<br>1 point: Intermediate risk<br>Aspirin at 100 mg<br>>1 point: High risk<br>Low molecular weight heparin at prophylactic dose or therapeutic dose of warfarin		

IMiD: immunomodulatory agent, MM: multiple myeloma, VTE: venous thromboembolism.

Adopted from: Fotiou D, Gavriatopoulou M, Terpos E. Multiple myeloma and thrombosis: prophylaxis and risk prediction tools. Cancers (Basel). 2020;12(1):191. doi: 10.3390/cancers12010191.
