# Supplementary material for: Nursing management of treatment-related venous thromboembolism in patients with multiple myeloma
Source: Front Med (Lausanne). 2023 Apr 18;10:1153694. doi: 10.3389/fmed.2023.1153694 (PMC10151651; doi:10.3389/fmed.2023.1153694)
Supplement: Supplementary file 2 [file Table_2.pdf]

Appendix 2 Clinical risk assessment models for VTE prediction in MM patients.

**CLINICAL RAMs for VTE in MM**	
**IMPEDE VTE Score**	**SAVED Score***
Immunomodulatory drug (+4)	Surgery (within the last 90 days) (+2)
BMI ≥ 25 kg/m^2 (+1)	Asian Race (−3)
Pathologic fracture pelvis/femur (+4)	VTE history (+3)
Erythropoiesis-stimulating agent (+1)	Eight (age >=80 years) (+1)
Dexamethasone (High-dose) (+4)	Dexamethasone dose<br>Standard (+1)
Dexamethasone Low-Dose (+2)	High (+2)
Doxorubicin (+3)	* for patients on IMiD-based regimens only
Ethnicity/Race = Asian (−3)	
VTE history (+5)	
Tunneled line/CVC (+2)	
Existing use of therapeutic warfarin or low molecular weight heparin (LWMH) (−5)	
Existing use of prophylactic LMWH or aspirin (−3)	
Stratified risk groups based on the weighted scoring system	
Low risk (score ≤3)	High risk (score (≥2)
Intermediate-risk (score of 4–7)	Low risk (≤1)
High risk (≥8 scores)	

RAM: risk assessment model; VTE: venous thromboembolism; MM: multiple myeloma; BMI: body mass index; CVC: central venous catheter; LMWH: low molecular weight heparin

Adopted from: Fotiou D, Gavriatopoulou M, Terpos E. Multiple myeloma and thrombosis: prophylaxis and risk prediction tools. Cancers (Basel). 2020;12(1):191. doi: 10.3390/cancers12010191.
